# Quality of child anthropometric data from SISVAN, Brazil, 2008-2017

**DOI:** 10.11606/s1518-8787.2023057004655

**Published:** 2023-09-14

**Authors:** Natanael de Jesus Silva, Juliana Freitas de Mello e Silva, Thaís Rangel Bousquet Carrilho, Elizabete de Jesus Pinto, Rafaella da Costa Santin de Andrade, Sara Araújo Silva, Jéssica Pedroso, Ana Maria Spaniol, Gisele Ane Bortolini, Andhressa Fagundes, Eduardo Augusto Fernandes Nilson, Rosemeire Leovigildo Fiaccone, Gilberto Kac, Maurício Lima Barreto, Rita de Cássia Ribeiro-Silva

**Affiliations:** I Fundação Oswaldo Cruz Instituto Gonçalo Moniz Centro de Integração de Dados e Conhecimentos para Saúde Salvador BA Brasil Fundação Oswaldo Cruz. Instituto Gonçalo Moniz. Centro de Integração de Dados e Conhecimentos para Saúde. Salvador, BA, Brasil.; II Universitat de Barcelona Instituto de Salud Global de Barcelona Barcelona España Universitat de Barcelona. Instituto de Salud Global de Barcelona. Barcelona, España.; III Universidade Federal do Rio de Janeiro Instituto de Nutrição Josué de Castro Observatório de Epidemiologia Nutricional Rio de Janeiro RJ Brasil Universidade Federal do Rio de Janeiro. Instituto de Nutrição Josué de Castro. Observatório de Epidemiologia Nutricional. Rio de Janeiro, RJ, Brasil.; IV Universidade Federal do Recôncavo da Bahia Santo Antônio de Jesus BA Brasil Universidade Federal do Recôncavo da Bahia. Santo Antônio de Jesus, BA, Brasil.; V Ministério da Saúde Coordenação-Geral de Alimentação e Nutrição Brasília DF Brasil Ministério da Saúde. Coordenação-Geral de Alimentação e Nutrição. Brasília, DF, Brasil.; VI Universidade Federal de Sergipe Departamento de Nutrição São Cristóvã SE Brasil Universidade Federal de Sergipe. Departamento de Nutrição. São Cristóvão, SE, Brasil.; VII Universidade Federal da Bahia Instituto de Matemática e Estatística Salvador BA Brasil Universidade Federal da Bahia. Instituto de Matemática e Estatística. Salvador, BA, Brasil.; VIII Universidade Federal da Bahia Instituto de Saúde Coletiva Salvador BA Brasil Universidade Federal da Bahia. Instituto de Saúde Coletiva. Salvador, BA, Brasil.; IX Universidade Federal da Bahia Escola de Nutrição Salvador BA Brasil Universidade Federal da Bahia. Escola de Nutrição. Salvador, BA, Brasil.

**Keywords:** Data Reliability, Food and Nutrition Surveillance, Health Information Systems, Anthropometry, Child

## Abstract

**OBJECTIVE:**

To evaluate the quality of anthropometric data of children recorded in the Food and Nutrition Surveillance System (SISVAN) from 2008 to 2017.

**METHOD:**

Descriptive study on the quality of anthropometric data of children under five years of age admitted in primary care services of the Unified Health System, from the individual databases of SISVAN. Data quality was annually assessed using the indicators: coverage, completeness, sex ratio, age distribution, weight and height digit preference, implausible z-score values, standard deviation, and normality of z-scores.

**RESULTS:**

In total, 73,745,023 records and 29,852,480 children were identified. Coverage increased from 17.7% in 2008 to 45.4% in 2017. Completeness of birth date, weight, and height corresponded to almost 100% in all years. The sex ratio was balanced and approximately similar to the expected ratio, ranging from 0.8 to 1. The age distribution revealed higher percentages of registrations from the ages of two to four years until mid-2015. A preference for terminal digits “zero” and “five” was identified among weight and height records. The percentages of implausible z-scores exceeded 1% for all anthropometric indices, with values decreasing from 2014 onwards. A high dispersion of z-scores, including standard deviations between 1.2 and 1.6, was identified mainly in the indices including height and in the records of children under two years of age and residents in the North, Northeast, and Midwest regions. The distribution of z-scores was symmetric for all indices and platykurtic for height/age and weight/age.

**CONCLUSIONS:**

The quality of SISVAN anthropometric data for children under five years of age has improved substantially between 2008 and 2017. Some indicators require attention, particularly for height measurements, whose quality was lower especially among groups more vulnerable to nutritional problems.

## INTRODUCTION

Anthropometry is universally used for nutritional surveillance of population groups^1,2^. Anthropometric data are periodically collected to provide a clear understanding of the magnitude and distribution of nutritional problems in a country, as well as to design and monitor interventions to improve the nutritional status of the population^3-5^. The availability of accurate prevalence estimates of stunting, wasting, overweight and obesity in children is essential to monitor local, national and global progress towards the goals of eradicating hunger and all forms of malnutrition^6^.

In Brazil, the monitoring of nutritional status is part of Food and Nutrition Surveillance (FNS), provided by the law that created the Brazilian Unified Health System (SUS), and consists of the continuous description of food and nutrition conditions of the Brazilian population^7^. The collection, recording and analysis of anthropometric data are routinely performed through population surveys by health professionals in primary care services, aimed at planning and organizing nutritional care and attention in SUS^7^. For anthropometric data to generate reliable information about the nutritional and health status of the local population, it is necessary to follow quality standards in the collection, recording and analysis of such data.

The quality of anthropometric data can be affected by multiple factors including sampling strategy, team training, measurement techniques and tools, non-response rate, data entry and processing methods^8-10^. Thus, several indicators have been proposed and used to assess the quality of such data, including population coverage^11,12^, completeness of birth date and anthropometric measurements^13,14^, preference for digits of age, height, and weight^15,16^, percentage of biologically implausible values^17^, as well as dispersion and distribution of standardized measures of weight and height^10,18^.

These indicators have been widely used to verify and control the quality of anthropometric data in population surveys and research, such as demographic and health surveys, in which they are used to account for the variability in data quality among different sites over time^10^. However, the application of these indicators has still been incipient to assess the quality of data routinely collected in health services. In Brazil, coverage indicators have been solely used to assess the quality of anthropometric data of the population assisted in SUS health services^11,12^.

Aiming to expand this approach, the objective of this study was to evaluate the quality of anthropometric data of children under five years of age recorded in the Food and Nutrition Surveillance System (SISVAN), a tool of the Ministry of Health to monitor the nutritional status of Brazilians served in Primary Health Care (PHC). This study covers the evaluation of multiple quality indicators, recommended by the Technical Expert Advisory group on nutrition Monitoring (TEAM) of the World Health Organization (WHO) and the United Nations Children’s Fund (UNICEF)^19^. This study is expected to guide those involved in FNS actions on how to improve the quality of anthropometric data in order to provide greater reliability to the metrics for local, state and national monitoring of the nutritional status of the Brazilian children population.

## METHOD

### Design of the Study

This is a descriptive study on the evaluation of the quality of anthropometric data of children aged 0 to 59 months, attended in PHC services in Brazil, in the period between 2008 and 2017. The information was obtained from the individual and anonymized SISVAN databases.

The raw data from SISVAN, made available for use in this project by the Center for Data and Knowledge Integration for Health (CIDACS), Oswaldo Cruz Foundation (Fiocruz), were used in accordance with institutional protocols for data security and privacy and as established by Resolution 466/2012 of the National Research Ethics Committee of the National Health Council. The project was submitted and approved by the ethics committee of the Institute of Collective Health of the Federal University of Bahia (CAAE: 41695415.0.0000.5030).

### Data Source

The SISVAN databases are composed of nutritional and food monitoring records from the PHC Health Information Systems. Regarding nutritional status, these databases include anthropometric data recorded in the *Bolsa Familia* Program (BFP) Management System, in e-SUS APS, and in SISVAN^20^. Data on the nutritional status monitoring of BFP beneficiaries, which occurs at least twice a year, are incorporated into SISVAN at the end of each BFP term (first term from January to June and second term from July to December). The records from the e-SUS APS are gradually incorporated into the Health Information System for Primary Care, respecting the schedule of data submission by health teams, and then exported to SISVAN after processing and validation of data^20^.

### Quality Indicators for Anthropometric Data

The quality of anthropometric data was assessed by means of multiple indicators, recommended by WHO-Unicef^19^: 1) completeness (coverage of the target population, completeness of date of birth, and completeness of anthropometric measurements); 2) sex ratio; 3) age distribution (histograms of age in years/months and month of birth; and index of dissimilarity for age in months); 4) digit preference of height and weight (histograms of terminal digits and whole numbers; and index of dissimilarity for terminal digits); 5) implausible z-score values (percentage of implausible z-scores); 6) standard deviation of z-scores; and 7) normality of z-scores (histograms, skewness, and kurtosis). To analyze the indicators, data on sex, date of birth, age (months and years), height (cm) and weight (kg) measurements were used, as well as the z-scores of the anthropometric indices commonly used to assess the nutritional status of children: Height/Age (H/A), Weight/Age (W/A), Weight/Height (W/H), and Body Mass Index/Age (BMI/A). Z-scores were calculated using the “STATA igrowup package” tool and the WHO child growth reference curves^21^. The estimated quality indicators are described in detail in Chart 1.

**Chart 1:** Description of the quality indicators of anthropometric data. Children 0-59 months of age. SISVAN, Brazil, 2008-2017

Quality indicator	Breakdown of indicator	Data used
Completeness		
Coverage of the target population, SUS users (%)	Number of children with nutritional status recorded in SISVAN divided by the number of children under 5 years old using the SUS, multiplied by 100. The population using the SUS was obtained by subtracting the total population (estimated by the IBGE) by the population covered by private health insurance (obtained from the ANS).	All children with nutritional status records in SISVAN. A single record per child each year (last follow-up date) was considered for the calculation of coverage.
Coverage of the total population, residents (%)	Number of children with nutritional status records in SISVAN divided by the total number of children under five years old (estimated by IBGE), multiplied by 100.	A single record per child each year (last follow-up date) was considered for the calculation of coverage.
Completeness of birth date (%)	Number of records with complete day, month, and year of birth divided by the total number of child records in SISVAN, multiplied by 100.	All nutritional status follow-up records in SISVAN.
Completeness of anthropometric measurements (%)	Number of records with completed weight and height measurements divided by the total number of child records in SISVAN, multiplied by 100.	All nutritional status follow-up records in SISVAN.
Sex ratio		
Sex ratio	Number of boys divided by the number of girls. The expected ratio is that represented by the national population.	All nutritional status follow-up records in SISVAN.
Age distribution		
Histograms of age (in years, months and month of birth)	Histograms were used to assess the pattern of age distribution in months, years, and month of birth. An approximately uniform distribution is expected. The age of the child was calculated from the date of birth and date of nutritional status monitoring.	All records with complete birth month and year.
Index of dissimilarity for age (in months)	The Myers “unblended” index was analyzed, according to the formula below, to identify the percentage of records deviating from a uniform distribution of age in months (0-59) $\frac{\sum_{i=1}^{60}\left|X_{is}-X_{ie}\right|}{2}$ in which Xis is the observed percentage and Xie is the expected percentage. The index ranges from 0 to 90, being 0 the optimum value.	All records with complete birth month and year.
Preference for height and weight digits		
Histograms of terminal digits and whole numbers for height (cm) and weight (kg)	Histograms were used to assess the pattern of distribution of terminal digits and whole numbers of height and weight. An approximately uniform distribution is expected.	All records with weight and/or height information.
Index of dissimilarity for terminal digits of height (cm) and weight (kg)	The Myers “unblended” index was analyzed, according to the formula below, to identify the percentage of records deviating from a uniform distribution for terminal digits of height and weight $\frac{\sum_{i=1}^{10}\left|X_{is}-X_{ie}\right|}{2}$ in which X_is_ is the observed percentage and X_ie_ is the expected percentage. The index ranges from 0 to 90, being 0 the optimum value.	All records with weight and/or height information.
Implausible z-score values		
Implausible z-scores for each anthropometric index (%)	Number of records with implausible z-score values divided by the total number of child records in SISVAN, multiplied by 100. Using the WHO macro “STATA igrowup package” flagging system, implausible z-score values were detected according to plausibility criteria (WHO, 1995): H/A (-6, +6), W/H (-5, +5), W/A (-6, +5) and BMI/A (-5, +5). Percentages above 1% is indicative of poor data quality.	All records with date of birth, weight and/or height information.
Dispersion of z-score values		
Standard deviation of plausible z-score values for each anthropometric index	Standard deviation was calculated using the following formula $\sqrt{\frac{\sum_{i=1}^{n}{\left(Y_{i}-\bar{Y}\right)}^{2}}{n-1}}$ in which n is the total number of observations, Yi is each value in the database, and *Y*ˉ is the mean of observations	All records with biologically plausible z-score values for the anthropometric index of interest.
Normality of z-score values		
Distribution of plausible z-score values for each anthropometric index	Kernel density plots were used to examine the distribution pattern of the z-score values for each index.	All records with biologically plausible z-score values for the anthropometric index of interest.
Skewness of plausible z-score values for each anthropometric index	Skewness was calculated by the Fisher-Pearson coefficient: $\frac{\sum_{i=1}^{n}{\left(Y_{i}-\bar{Y}\right)}^{3}/n}{s^{3}}$ in which *Y*ˉ = mean, s = standard deviation (calculated with ‘n’ in the denominator instead of n-1) and n = sample size. It is generally accepted that a coefficient <-0.5 or >0.5 indicates skewness	All records with biologically plausible z-score values for the anthropometric index of interest.
Kurtosis of plausible z-score values for each anthropometric index	Kurtosis was calculated by the Fisher-Pearson coefficient: $\frac{\sum_{i=1}^{n}{\left(Y_{i}-\bar{Y}\right)}^{4}/n}{s^{4}}$ in which *Y*ˉ = mean, s = standard deviation (calculated with ‘n’ in the denominator instead of n-1) and n = sample size. In general, it is accepted that a coefficient <2 or >4 indicates kurtosis.	All records with biologically plausible z-score values for the anthropometric index of interest.

SISVAN: Food and Nutrition Surveillance System; SUS: Unified Health System; IBGE: Brazilian Institute of Geography and Statistics; ANS: National Supplementary Health Agency; H/A: Height/Age; W/H: Weight/Height; W/A: Weight/Age; BMI/A: Body Mass Index/Age.

### Data Processing and Analysis

Data were analyzed using Stata software version 15.1 (Stata Corporation, College Station, USA). Since the same record can be entered in the different information systems that comprise SISVAN, duplicate records were identified and removed considering the following variables: identification code of the individual, date of birth, date of follow-up, weight, and height. Duplicate records were accepted when all values of considered variables were equal. Quality indicators were annually described and according to the following variables when applicable: sex, age, region, and federative unit.

## RESULTS

### Target Population Coverage

In total, 73,745,023 records and 29,852,480 children under five years of age were identified in the SISVAN nutritional status databases between 2008 and 2017, after excluding 27,167,791 duplicate records. The coverage of the target SUS-using population increased from 17.7% in 2008 to 45.4% in 2017 (Table 1). The coverage of the total (resident) population increased from 14.3% to 34.6% in that same period. The increase in coverage was observed in all regions, with emphasis on the Northeast, where it was the highest in all years and the only region to reach more than 50% of the target population. Among the federative units, the highest coverage was observed in Minas Gerais (71.6% in 2017), Paraíba (69.7%), Piauí (69.4%), Ceará (58.9%), and Maranhão (58.9%). In 2017, only 11 federative units had coverage greater than 50% (Table 2).

**Table 1:** Quality indicators of anthropometric data. Children 0-59 months of age. SISVAN, Brazil, 2008-2017.

Indicators	2008	2009	2010	2011	2012	2013	2014	2015	2016	2017
Total of registrations and followed-up children										
Total number of children	2,172,824	2,752,361	3,339,585	3,263,701	3,189,390	4,216,064	4,343,156	4,943,025	5,134,115	5,097,377
Total of unique records	3,612,015	5,134,933	5,987,400	6,446,453	6,252,667	7,894,213	7,822,944	9,756,986	10,357,127	10,480,285
Total of duplicate records	1,969,446	2,908,094	2,950,561	2,888,367	2,595,380	2,287,606	2,197,925	2,988,357	3,172,167	3,209,888
Completeness										
Coverage of the target population, SUS users (%)	17.7	22.9	28.8	28.6	28.6	38.5	39.9	44.7	45.9	45,4
Coverage of the total population, resident (%)	14.3	18.3	22.5	22.2	21.8	28.9	29.8	33.7	34.9	34,6
Completeness of birth date (%)	99.9	99.9	99.9	99.9	99.9	99.9	99.9	100	100	100
Completeness of anthropometric measurements (%)	100	99.9	100	100	100	100	100	100	98.8	100
Sex ratio										
Sex ratio	1	1	1	1	0.8	0.8	0.9	1	1	1
Age distribution										
Dissimilarity Index for age in months (%)	20.4	14.1	12.6	11.9	11.2	10.3	9.7	4.8	1.5	3.8
Preference for height and weight digits										
Index of dissimilarity for terminal digits of height (%)	88.8	88	88.2	87.9	87.7	89.7	89.9	89.4	88.3	88.8
Index of dissimilarity for terminal digits of weight (%)	50.2	47	47.6	46.1	44.3	45.3	44.2	42.8	40.1	40.4
Implausible z-score values										
Implausible z-scores of H/A (%)	3	3.4	3.8	3.4	3	3.3	3.5	3	2.9	1.9
Implausible z-scores of W/H (%)	2.4	2.4	2.7	2.5	2.5	3.3	3.2	3	2.4	2.2
Implausible z-scores of W/A(%)	2.1	1.6	1.9	1.8	1.7	2.5	2.4	2.1	1.2	0.8
Implausible z-scores of BMI/A (%)	3.9	4.1	4.6	4.2	3.9	4.8	4.8	4.2	3.1	2.9
Dispersion of z-score values										
Standard Deviation of H/A z-scores	1.5	1.6	1.6	1.6	1.6	1.6	1.6	1.6	1.6	1.6
Standard deviation of W/H z-scores	1.3	1.4	1.5	1.5	1.5	1.5	1.5	1.5	1.5	1.5
Standard deviation of W/A z-scores	1.2	1.3	1.3	1.3	1.3	1.3	1.3	1.3	1.3	1.3
Standard deviation of BMI/A z-scores	1.4	1.5	1.5	1.5	1.5	1.5	1.5	1.5	1.5	1.5
Normality of z-score values										
Skewness of H/A z-scores	0.1	0.2	0.2	0.2	0.1	0.2	0.2	0.1	0.1	0.1
Skewness of W/H z-scores	0	-0.1	-0.1	-0.1	-0.1	-0.1	-0.1	-0.1	0	0
Skewness of W/A z-scores	0	0.1	0.1	0.1	0.1	0.1	0.1	0.1	0.1	0.1
Skewness of BMI/A z-scores	-0.1	-0.1	-0.1	-0.1	-0.1	-0.1	-0.1	-0.1	-0.1	-0.1
Kurtosis of H/A z-scores	4.7	4.5	4.5	4.5	4.5	4.5	4.5	4.4	4.5	4.5
Kurtosis of W/H z-scores	3.9	3.8	3.8	3.8	3.8	3.7	3.7	3.7	3.7	3.7
Kurtosis of W/A z-scores	4.3	4.1	4.1	4.1	4.1	4.1	4.1	4.1	4.1	4.1
Kurtosis of BMI/A z-scores	3.9	3.8	3.7	3.7	3.7	3.7	3.7	3.7	3.7	3.7

SISVAN: Food and Nutrition Surveillance System; H/A: Height/Age; W/H: Weight/Height; W/A: Weight/Age; BMI/A: Body Mass Index/Age.

**Table 2:** Coverage of the target population and total population, by region and federative unit. Children 0-59 months of age. SISVAN, Brazil.

Regions and states	Target population (SUS user)		Total population (resident)	
	
	2008	2017	2008	2017
North	15.5	47.8	14.3	43.2
Acre	11.7	49.9	11.3	47.9
Amapá	8.4	31	7.7	28.5
Amazonas	15.7	51.1	14	45.5
Pará	15.3	50	14.1	45
Rondônia	15	29.7	14.1	26.9
Roraima	16.6	35.2	15.8	33.5
Tocantins	22.5	58.8	21.5	54.3
Northeast	23.2	56.7	21.3	49.1
Alagoas	23.6	56.9	21.8	49.4
Bahia	23.2	54.7	21.4	48.6
Ceará	27.9	58.9	25.1	48.3
Maranhão	21.8	58.9	21.1	54.7
Paraíba	32.9	69.7	30.4	60.9
Pernambuco	16.5	49.4	14.5	41.5
Piauí	24.8	69.4	23.2	59.8
Rio Grande do Norte	23.5	47.6	20.6	39.3
Sergipe	17.4	55.3	15.7	46.3
Midwest	10.5	30.7	9.2	24.2
Distrito Federal	0.9	13.3	0.7	9.7
Goiás	12.6	27.9	11.3	22.3
Mato Grosso	11	38.6	10	31.1
Mato Grosso do Sul	14.1	41.8	12.3	33
Southeast	14.2	38.5	9.5	25.1
Espírito Santo	24.1	31.5	18.7	23.4
Minas Gerais	19.5	71.6	15.5	51.7
Rio de Janeiro	9.7	26.8	6.6	17.8
São Paulo	11.6	27.1	7	16.5
South	17.8	42.3	14.2	31.4
Paraná	19.9	48	15.9	34.6
Rio Grande do Sul	16.3	35.6	13.1	26.7
Santa Catarina	16.4	43.3	13.1	33.2
Brasil	17.7	45.4	14.3	34.6

SISVAN: Food and Nutrition Surveillance System. SUS: Unified Health System.

### Completeness of Date of Birth and Anthropometric Measurements

The percentage of records with complete birth date in SISVAN was high for the entire period studied, ranging from 99.9% in 2008 to 100% in 2017 (Table 1). The percentage of records with completed weight and height measurements was also high, showing 100% in almost all years (Table 1).

### Sex Ratio

The sex ratio ranged between 0.8 and 1 over the years. Variability in this indicator was identified among 2012 and 2014, when ratios showed higher numbers of girls compared to boys (Table 1).

### Age Distribution

**Figure 1:**
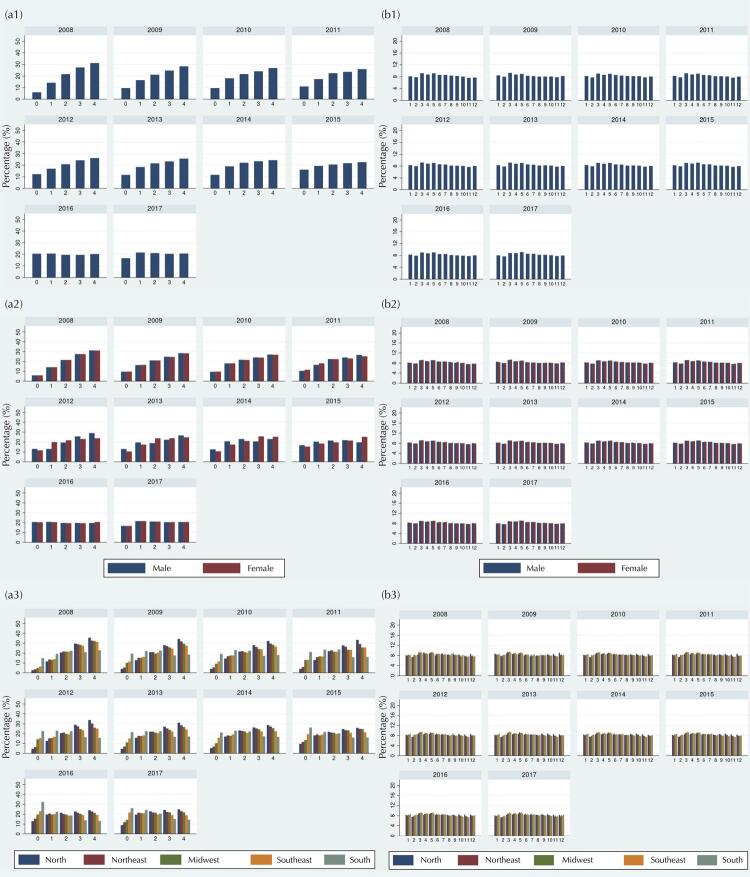
Distribution according to age in years (a) and month of birth (b) in the total population (1) and according to sex (2) and region (3). Children 0-59 months of age. SISVAN, Brazil, 2008-2017.

Histograms of age in completed years revealed a pattern of higher percentages of registrations among ages two to four years until mid-2015, when the age distribution became more uniform (Figure 1a). Such a pattern was observed mainly in the North and Northeast regions. Birth months showed approximately uniform distribution regardless of year, sex, and region (Figure 1b). According to the index of dissimilarity, the percentage of records to be redistributed to obtain a uniform distribution of age in months reduced from 20.4% in 2008 to 3.8% in 2017 (Table 1).

### Digit Preference for Height and Weight

**Figure 2:**
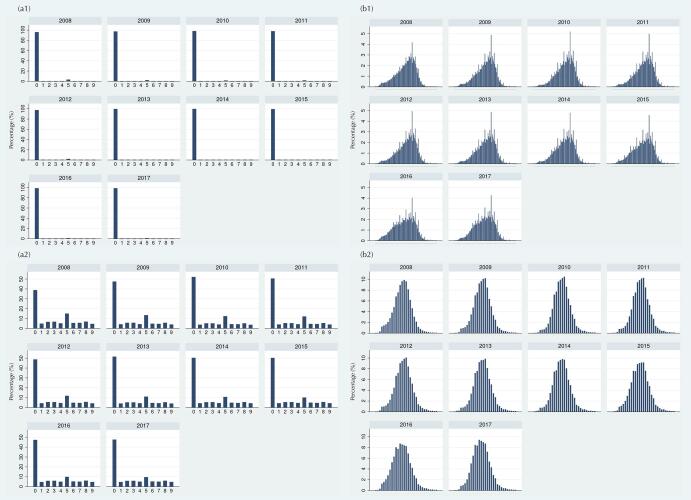
Preference for terminal digits (a) and for whole numbers of (b) height in cm (1) and weight in kg (2). Children 0-59 months of age. SISVAN, Brazil, 2008-2017.

Histograms in Figure 2a show almost 100% of preferences for the terminal digit zero for height, and terminal digits zero and five for weight. Almost 90% of height records (largest possible number) would need to be redistributed to obtain an uniform distribution of terminal digits across all years; while for terminal digits of weight, the percentage of records that would need to be redistributed reduced from 50.2% in 2008 to 40.4% in 2017 (Table 1). The distribution of whole numbers showed several noticeable peaks for height measurement (e.g., 100 cm) (Figure 2a), while the distribution of whole numbers for weight was very adequate, showing no peaks (Figure 2b).

* The whole numbers represented in the x-axis (horizontal) of the graphics include 40–140 cm for height (b.1) and ≤ 30 kg for weight (b.2).

### Implausible Z-score Values

**Figure 3:**
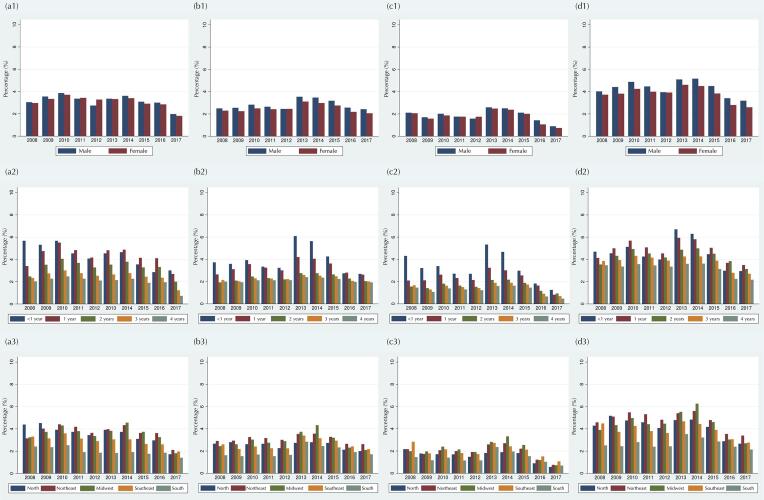
Percentage of the implausible z-scores values for height/age (a), weight/height (b), weight/age (c), and BMI/age (d) according to sex (1), age in completed years (2), and region of residence (3). Children 0-59 months of age. SISVAN, Brazil, 2008-2017.

The percentages of implausible z-scores according to WHO cutoff points varied until mid-2014, when a decrease was observed in the following years for all anthropometric indices (Table 1): H/A (3.5% in 2014 vs. 1.9% in 2017), W/H (3.2% vs. 2.2%), W/A (2.4% vs. 0.8%), and BMI/A (4.8% vs. 2.9%). The highest percentages of implausibility were identified among records for children under two years old and from the North, Northeast, and Midwest regions (Figure 3).

### Dispersion of Z-score Values

**Figure 4:**
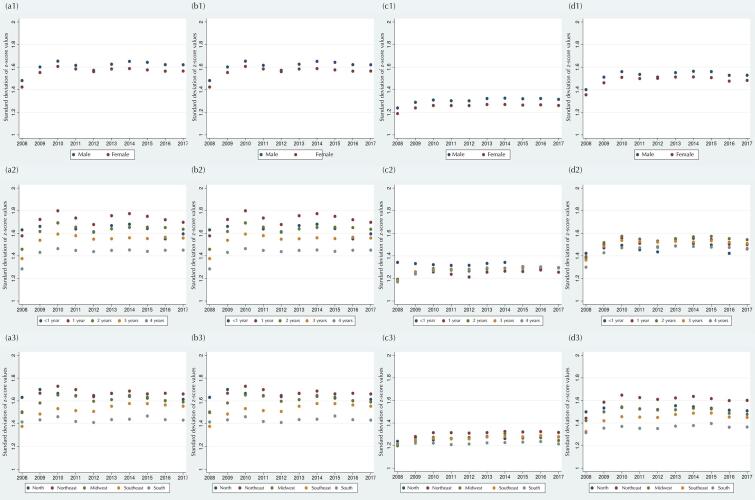
Standard deviation of the z-scores for height/age (a), weight/height (b), weight/age (c), and BMI/age (d) according to sex (1), age in completed years (2), and region of residence (3). Children 0-59 months of age. SISVAN, Brazil, 2008-2017.

Standard deviation values greater than 1 for plausible z-score measures were found for all anthropometric indices throughout the period (Table 1). The lowest standard deviation values were observed in 2008. A stability in the dispersion of z-scores for all indices was noticed after 2008: H/A (1.6), W/H (1.5), W/A (1.3) and BMI/A (1.5). In general, there was greater dispersion of z-score values among children two years of age or younger and residents in the Northeast, North, and Midwest regions (Figure 4).

### Normality of Z-score Values

**Figure 5:**
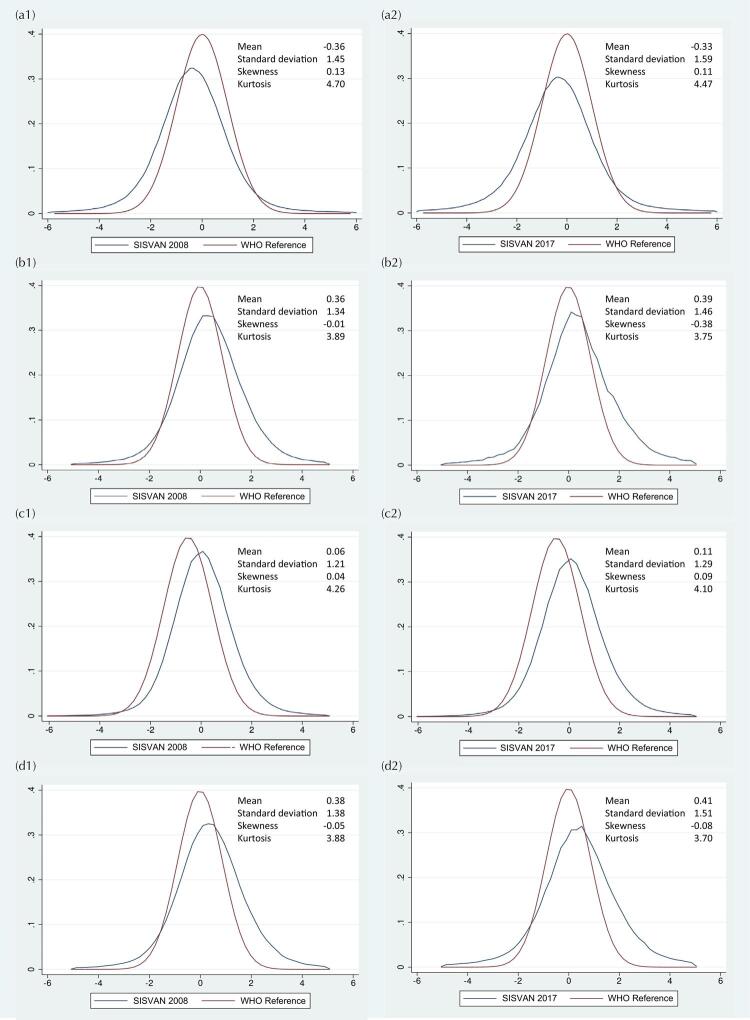
Kernel density graphs of the z-scores for height/age (a), weight/height (b), weight/age (c), and BMI/age (d), in 2008 (1) and 2017 (2). Children 0-59 months of age. SISVAN, Brazil, 2008-2017.

The distribution curves of z-scores in 2008 and 2017 showed flattening and leftward deviation for H/A, and more modest flattening and rightward deviations were observed in the distributions of z-scores of W/H, W/A, and BMI/I, compared to the normal distribution pattern of WHO child growth curves (Figure 5). According to Fisher-Pearson coefficient values, the distribution of z-scores was symmetric for all indices, whereas the distributions of H/A and W/A z-scores were platykurtic in all years, which is explained by coefficients above 4 and flattening of the curve (Table 1).

## DISCUSSION

This study examined the quality of anthropometric data of children seen in PHC between 2008 and 2017. It is the first one assessing the quality of individual SISVAN data in Brazil, covering multiple indicators and different dimensions. Overall, the results show that the quality of anthropometric data collected and recorded in PHC information systems has improved substantially over the years. These findings represent a milestone for the consolidation of SISVAN, allowing better clarity and reliability in the use and analysis of data to identify nutritional problems in the population and for decision making in food and nutrition policies.

Completeness is one of the dimensions of data quality that is directly related to selection biases and, consequently, to the representativeness of the results. High completeness was observed throughout the period in date of birth and anthropometric measurements, which are mandatory information for registration. The results also showed a growing expansion of SISVAN coverage over the years, especially among the SUS user population. This expansion can be attributed to important advances: the successful articulation of FNS actions with other education and social assistance policies (for example, the School Health Program and the BFP^7^); expansion of the coverage of community health agents and family health teams11; expansion and qualification of PHC, through the Family Health Support Teams and the Primary Health Care Access and Quality Improvement Program (PMAQ-AB)7; and investment in FNS actions through the Financing for Food and Nutrition Actions (FNA) and financial support for municipalities to purchase appropriate anthropometric equipment for primary care^22^.

Despite the clear progress over the years, the coverage of SISVAN still remains incipient in most regions and federative units of the country. In 2017, only 11 out of the 27 federative units had coverage above 50%. Also, nine of the 11 federative units are from the Northeast region, which concentrates the largest number of BFP beneficiaries^23^. Previous studies show that about 80% of anthropometric records incorporated by SISVAN, between 2008 and 2013, came from the BFP conditionalities^11^, which include nutritional monitoring of children under seven years of age, reflecting in higher coverage of SISVAN for the most socioeconomically vulnerable Brazilian population. It is worth noting that, due to the Covid-19 pandemic and its effects on health services, a substantial drop in BFP conditionalities monitoring was detected in 2020, since of the 7.3 million beneficiary children, only two million had follow-up records, which corresponds to coverage of only 25.5%^24^.

Another important aspect related to the external validity and representativeness of the data is the distribution of the population according to sex and age. The sex ratio found in SISVAN was balanced and approximately similar to that expected for the Brazilian population under five years of age (1.05)^25^. The age distribution in complete years and months did not show substantial peaks. However, a pattern of higher percentages of records between the ages of two to four years was observed until mid-2015, when the age distribution became more uniform. This pattern was observed mainly in the North and Northeast regions. As these regions concentrate the largest number of records in SISVAN, coming from the BFP^11^, part of the distribution of this age can be explained by the late inclusion and monitoring of children by the program.

The preference for height and weight digits may signal from the rounding of measurements to the use of inadequate equipment and care during data collection and recording. A preference for the terminal digits zero and five was observed in measurements of height and weight, indicating systematic error by the rounding of measurements. Among whole numbers, several noticeable peaks for height measurements were observed (e.g., 100 cm), revealing possible problems with equipment or rounding of measurements. On the other hand, the distribution of whole numbers for weight was very adequate.

Although critical to obtaining accurate prevalence of nutritional status, these results are relatively common and expected, especially for height measurements^8^. In most anthropometric rulers, centimeter marks are larger and easier to read than millimeter marks, inducing less diligent or less knowledgeable staff to record rounded values. As observed in the SISVAN data, rounding of weight measurements was less common, possibly due to the use of digital scales whose displays provide numerical values with easy-to-read decimals.

It is also worth mentioning the adequacy of structures and equipment for collecting these data in basic health units. According to a recent study, based on data from the external evaluation of PMAQ-AB in 2014, only 35% of primary health units in Brazil had an adequate structure for the development of FNS, including adult and child scales, anthropometric ruler, measuring tape, and child health booklet^26^.

The implausibility, dispersion, and normality indicators of z-scores are usually associated with measurement errors, inaccurate date of birth, or errors in data recording. Despite the reduction of implausible values among SISVAN records from 2014, the percentages still exceeded 1% for all indices, suggesting low data quality according to the WHO implausibility system^2,19^. A similar result was found for the z-score dispersion indicator. Although values of standard deviation remained stable over the years, a large dispersion of z-scores was noted for most indices. Previous studies have reported wide variation in the standard deviation of anthropometric z-scores in children under five years of age in demographic and health surveys in several low- and middle-income countries^10,18^.

Consistent patterns of higher percentages of implausibility and dispersion of z-scores were observed among anthropometric indices including height measurement, and records of children under two years of age and residents of the North, Northeast, and Midwest regions. Such results point to well-known errors and limitations on the collection and recording of anthropometric measurements. It is generally expected that the standard deviation of H/A and BMI/A is higher than that of the other indices, due to the greater difficulty and chance of errors in collecting height and age measurements.

This pattern is especially expected in the group of children younger than two years of age, whose height is measured with them lying down and the accuracy of age in months is more critical due to the faster growth rate in this age group^(19,27)^. Furthermore, it is noteworthy that regions where the standard deviation and percentage of implausible values were higher are the most vulnerable from the point of view of adequate structure for nutritional surveillance in primary health units, according to a study with data from PMAQ-AB^26^.

Different parameters and normality measures were used to assess the distribution of z-scores for each anthropometric index. From the Kernel density plots, we observed distribution deviations to the left for H/A, and to the right for W/H, W/A and BMI/A, as compared to the normal distribution pattern of the WHO growth curves. Although the z-score distributions were symmetric for the four indices, kurtic distributions were identified for H/A and W/A (Fisher-Pearson coefficient > 4).

Despite these findings, there is still no consistent evidence to suggest that the dispersion and deviation from a Gaussian distribution is due to data quality alone. The WHO reference population used to derive the z-scores was restricted to a healthy population living in favorable environmental conditions for healthy growth^28^. Thus, it is possible that unusual distributions may occur in more heterogeneous populations, such as in countries with large social inequalities. The SISVAN population represents the users of PHC in Brazil, composed mostly of BFP beneficiaries; i.e., a more socioeconomically vulnerable population. The distributions found in this study are consistent with estimates of malnutrition in this population, which reveal persistent prevalence of short stature and increasing burden of overweight and obesity in children^29,30^.

This study has some limitations. Interpretation of certain indicators alone may not be sufficient to draw conclusions on the quality of the data, especially for indicators that take into account the dispersion and distribution of z-scores. More research is needed to quantify in definitive terms how much of the distribution of z-scores is attributable to population heterogeneity or measurement error. In the absence of cut-off points or more appropriate approaches that consider such limitations, a joint assessment of quality indicators is recommended^19^.

Based on the results of this study, we highlight the importance of actions to improve critical points identified in the quality of anthropometric data from SISVAN: 1) maintenance and expansion of intersectoral policies and health programs that promote FNS actions, as occur in the BFP, School Health Program and Growing Healthy Program; 2) development of qualification and continuing education actions (preferably attentive and flexible to the different local realities); 3) maintenance and expansion of financial support to municipalities for structuring FNS in PHC, through the acquisition and periodic calibration of anthropometric equipment; 4) computerization in PHC services, allowing professionals in primary health units to promptly record data on care, including weight and height data, in the patient’s electronic medical record in e-SUS APS; and 5) implantation and implementation of routine for continuous verification and production of reports on the quality of data in SISVAN.

## CONCLUSION

Overall, the results suggest that the quality of anthropometric data in SISVAN has substantially improved over the years. However, some indicators still require attention. The coverage of the target population remains incipient for a surveillance system whose objective is the universal monitoring of the public that uses SUS primary care. The accuracy and quality of anthropometric measurements, especially of height, were lower in records of children under two years of age and residents in North, Northeast, and Midwest regions. Such groups are the portion of the child population most vulnerable to nutritional problems, requiring accurate estimates that can support the monitoring of the population nutritional profile and the development of public policies.

## References

[1] Gorstein J, Akré J (1988). The use of anthropometry to assess nutritional status. World Health Stat Q.

[2] World Health Organization (1995). Physical status: the use and interpretation of anthropometry.

[3] Abarca-Gómez L, Abdeen ZA, Hamid ZA, Abu-Rmeileh NM, Acosta-Cazares B, Acuin C (2017). Worldwide trends in body-mass index, underweight, overweight, and obesity from 1975 to 2016: a pooled analysis of 2416 population-based measurement studies in 128·9 million children, adolescents, and adults. Lancet.

[4] Afshin A, Forouzanfar MH, Reitsma MB, Sur P, Estep K, Lee A (2017). Health effects of overweight and obesity in 195 countries over 25 years. N Engl J Med.

[5] Rodriguez-Martinez A, Zhou B, Sophiea MK, Bentham J, Paciorek CJ, Iurilli ML (2020). Height and body-mass index trajectories of school-aged children and adolescents from 1985 to 2019 in 200 countries and territories: a pooled analysis of 2181 population-based studies with 65 million participants. Lancet.

[6] World Health Organization (2017). Global nutrition monitoring framework: operational guidance for tracking progress in meeting targets for 2025.

[7] Ministério da Saúde (BR) (2015). Marco de referência da vigilância alimentar e nutricional na atenção básica.

[8] Bagni UV, Barros DC (2015). Erro em antropometria aplicada à avaliação nutricional nos serviços de saúde: causas, consequências e métodos de mensuração. Nutrire.

[9] Corsi DJ, Perkins JM, Subramanian SV (2017). Child anthropometry data quality from demographic and health surveys, multiple indicator cluster surveys, and national nutrition surveys in the West Central Africa region: are we comparing apples and oranges?. Glob Health Action.

[10] Perumal N, Namaste S, Qamar H, Aimone A, Bassani DG, Roth DE (2020). Anthropometric data quality assessment in multisurvey studies of child growth. Am J Clin Nutr.

[11] Nascimento FA, Silva SA, Jaime PC (2017). Coverage of assessment of nutritional status in the Brazilian Food and Nutritional Surveillance System, 2008-2013. Cad Saude Publica.

[12] Mourão E, Gallo CO, Nascimento FA, Jaime PC (2020). Temporal trend of Food and Nutrition Surveillance System coverage among children under 5 in the Northern Region of Brazil, 2008-2017. Epidemiol Serv Saude.

[13] Finaret AB, Hutchinson M (2018). Missingness of height data from the demographic and health surveys in Africa between 1991 and 2016 was not random but is unlikely to have major implications for biases in estimating stunting prevalence or the determinants of child height. J Nutr.

[14] Nannan N, Dorrington R, Bradshaw D (2019). Estimating completeness of birth registration in South Africa, 1996-2011. Bull World Health Organ.

[15] Lyons-Amos M, Stones T (2017). Trends in demographic and health survey data quality: an analysis of age heaping over time in 34 countries in Sub Saharan Africa between 1987 and 2015. BMC Res Notes.

[16] Bopp M, Faeh D (2008). End-digits preference for self-reported height depends on language. BMC Public Health.

[17] Lawman HG, Ogden CL, Hassink S, Mallya G, Vander Veur S, Foster GD (2015). Comparing methods for identifying biologically implausible values in height, weight, and body mass index among youth. Am J Epidemiol.

[18] Mei Z, Grummer-Strawn LM (2007). Standard deviation of anthropometric Z-scores as a data quality assessment tool using the 2006 WHO growth standards: a cross country analysis. Bull World Health Organ.

[19] World Health Organization, United Nations Children’s Fund (2019). Recommendations for data collection, analysis and reporting on anthropometric indicators in children under 5 years old.

[20] Ministério da Saúde (BR) (2017). Manual operacional para uso do sistema de vigilância alimentar e nutricional: SISVAN VERSÃO 3.0.

[21] World Health Organization (2020). WHO Anthro Survey Analyzer and other tools.

[22] Ministério da Saúde (BR) (2011). Portaria nº 2975, de 14 de dezembro de 2011. Apoiar financeiramente a estruturação da Vigilância Alimentar e Nutricional.

[23] Ministério da Cidadania (BR), Secretaria de Avaliação e Gestão da Informação (2020). VIS DATA 3 beta. Quantidade total de pessoas em famílias beneficiárias do Programa Bolsa Família.

[24] Ministério da Cidadania (BR) (2020). Resultado do acompanhamento das condicionalidades de saúde no 1º semestre de 2020.

[25] Instituto Brasileiro de Geografia e Estatística, Sistema IBGE de Recuperação Automática - Sidra Projeção da população: tabela 7358 - População, por sexo e idade.

[26] Machado PM, Lacerda JT, Colussi CF, Calvo MC (2021). Estrutura e processo de trabalho para as ações de alimentação e nutrição na Atenção Primária à Saúde no Brasil, 2014. Epidemiol Serv Saude.

[27] Larsen AF, Headey D, Masters WA (2019). Misreporting month of birth: diagnosis and implications for research on nutrition and early childhood in developing countries. demography.

[28] World Health Organization, Multicentre Growth Reference Study Group (2006). Enrolment and baseline characteristics in the WHO Multicentre Growth Reference Study. Acta Paediatr Suppl.

[29] Ribeiro-Silva RC, Silva NJ, Felisbino-Mendes MS, Falcão IR, Andrade RD, Silva SA (2021). Time trends and social inequalities in child malnutrition: nationwide estimates from Brazil’s food and nutrition surveillance system, 2009-2017. Public Health Nutr.

[30] Silva NJ, Ribeiro-Silva RC, Rasella D, Alves FJ, Campello T, Fiaccone RL (2021). Shifts towards overweight and double burden of malnutrition among socio-economically vulnerable children: a longitudinal ecological analysis of Brazilian municipalities. Public Health Nutr.

