# Supplementary material for: Quality of child anthropometric data from SISVAN, Brazil, 2008-2017
Source: Rev Saude Publica. 2023 Sep 14;57:62. doi: 10.11606/s1518-8787.2023057004655 (PMC10519688; doi:10.11606/s1518-8787.2023057004655)
Supplement: Translation

# Translation: Qualidade dos dados antropométricos infantis do Sisvan, Brasil, 2008-2017

**Authors:** Natanael de Jesus Silva, Juliana Freitas de Mello e Silva, Thaís Rangel Bousquet Carrilho, Elizabete de Jesus Pinto, Rafaella da Costa Santin de Andrade, Sara Araújo Silva, Jéssica Pedroso, Ana Maria Spaniol, Gisele Ane Bortolini, Andhressa Fagundes, Eduardo Augusto Fernandes Nilson, Rosemeire Leovigildo Fiaccone, Gilberto Kac, Maurício Lima Barreto, Rita de Cássia Ribeiro-Silva

**Affiliations:** I Brasil Fundação Oswaldo Cruz. Instituto Gonçalo Moniz. Centro de Integração de Dados e Conhecimentos para Saúde. Salvador, BA, Brasil.; II España Universitat de Barcelona. Instituto de Salud Global de Barcelona. Barcelona, España.; III Brasil Universidade Federal do Rio de Janeiro. Instituto de Nutrição Josué de Castro. Observatório de Epidemiologia Nutricional. Rio de Janeiro, RJ, Brasil.; IV Brasil Universidade Federal do Recôncavo da Bahia. Santo Antônio de Jesus, BA, Brasil.; V Brasil Ministério da Saúde. Coordenação-Geral de Alimentação e Nutrição. Brasília, DF, Brasil.; VI Brasil Universidade Federal de Sergipe. Departamento de Nutrição. São Cristóvão, SE, Brasil.; VII Brasil Universidade Federal da Bahia. Instituto de Matemática e Estatística. Salvador, BA, Brasil.; VIII Brasil Universidade Federal da Bahia. Instituto de Saúde Coletiva. Salvador, BA, Brasil.; IX Brasil Universidade Federal da Bahia. Escola de Nutrição. Salvador, BA, Brasil.

**Keywords:** Confiabilidade dos Dados, Vigilância Alimentar e Nutricional, Sistemas de Informação em Saúde, Antropometria, Criança

## Abstract

**OBJETIVOS:**

Avaliar a qualidade dos dados antropométricos de crianças registradas no Sistema de Vigilância Alimentar e Nutricional (Sisvan) no período 2008-2017.

**MÉTODOS:**

Estudo descritivo sobre a qualidade dos dados antropométricos de crianças menores de 5 anos atendidas nos serviços de atenção primária do Sistema Único de Saúde, a partir das bases de dados individuais do Sisvan. A qualidade dos dados foi avaliada anualmente por meio dos indicadores: cobertura, completude, razão entre sexos, distribuição da idade, preferência por dígitos de peso e estatura, valores de escore-z implausíveis, desvio-padrão e normalidade dos escores-z.

**RESULTADOS:**

No total, 73.745.023 registros e 29.852.480 crianças foram identificados. A cobertura aumentou de 17,7% em 2008 para 45,4% em 2017. A completude da data de nascimento, peso e estatura correspondeu a quase 100% para todos os anos. A razão entre sexos foi equilibrada e aproximadamente similar a razão esperada, variando entre 0,8 e 1. A distribuição da idade revelou maiores percentuais de registros entre as idades de 2 a 4 anos até meados de 2015. Uma preferência pelos dígitos terminais “zero” e “cinco” foi identificada entre os registros de peso e estatura. As porcentagens de escores-z implausíveis excederam 1% para todos os índices antropométricos, com redução dos valores a partir de 2014. Uma alta dispersão dos escores-z, incluindo desvios-padrão entre 1,2 e 1,6, foi identificada principalmente nos índices incluindo estatura e nos registros de crianças menores de 2 anos e residentes das regiões Norte, Nordeste e Centro-Oeste. A distribuição dos escores-z foi simétrica para todos os índices e platicúrtica para estatura/idade e peso/idade.

**CONCLUSÕES:**

A qualidade dos dados antropométricos do Sisvan para crianças menores de 5 anos melhorou substancialmente entre 2008 e 2017. Alguns indicadores requerem atenção, sobretudo para medidas de estatura, cuja qualidade foi principalmente inferior entre os grupos mais vulneráveis a agravos nutricionais.

## INTRODUÇÃO

A antropometria é universalmente utilizada para a vigilância nutricional de grupos populacionais^1,2^. Dados antropométricos são periodicamente coletados para fornecer um entendimento claro da magnitude e distribuição dos problemas nutricionais em um país e para projetar e monitorar intervenções com o propósito de melhorar o estado nutricional da população^3^. A disponibilidade de estimativas precisas da prevalência de déficit de crescimento, baixo peso, sobrepeso e obesidade na população infantil é fundamental para monitorar o progresso local, nacional e global em direção às metas de erradicação da fome e de todas as formas de má nutrição^6^.

No Brasil, o monitoramento do estado nutricional é parte da Vigilância Alimentar e Nutricional (VAN), prevista na lei que cria o Sistema Único de Saúde (SUS), e consiste na descrição contínua das condições de alimentação e nutrição da população brasileira^7^. A coleta, registro e análise de dados antropométricos são realizados por meio de inquéritos populacionais e rotineiramente pelos profissionais de saúde nos serviços de atenção primária, visando o planejamento e organização do cuidado e da atenção nutricional no SUS^7^. Para que os dados antropométricos gerem informações fidedignas sobre o estado nutricional e sobre a situação de saúde da população local, é necessário seguir padrões de qualidade para coleta, registro e análise de tais dados.

A qualidade dos dados antropométricos pode ser afetada por múltiplos fatores que incluem a estratégia de amostragem, treinamento da equipe, técnicas e ferramentas de medição, taxa de não resposta, métodos de entrada e processamento dos dados^8^. Nesse sentido, vários indicadores têm sido propostos e utilizados para avaliação da qualidade desses dados, incluindo cobertura populacional^11,12^, completude da data de nascimento e das medidas antropométricas^13,14^, preferência por dígitos de idade, altura e peso^15,16^, percentual de valores biologicamente implausíveis^17^, bem como dispersão e distribuição das medidas padronizadas de peso e altura^10,18^.

Esses indicadores têm sido amplamente utilizados para verificação e controle de qualidade dos dados antropométricos de inquéritos e pesquisas populacionais, a exemplo das pesquisas de demografia e saúde, nas quais são usados para contabilizar a variabilidade na qualidade dos dados entre diferentes locais e ao longo do tempo^10^. Entretanto, a aplicação desses indicadores ainda tem sido muito incipiente para avaliar a qualidade de dados coletados rotineiramente em serviços de saúde. No Brasil, indicadores de cobertura têm sido unicamente utilizados para avaliar a qualidades dos dados antropométricos da população atendida em serviços de saúde do SUS^11,12^.

Com vistas à ampliação dessa abordagem, o objetivo deste estudo foi avaliar a qualidade dos dados antropométricos de crianças menores de 5 anos registradas no Sistema de Vigilância Alimentar e Nutricional (Sisvan), ferramenta do Ministério da Saúde para monitorar o estado nutricional dos brasileiros atendidos na Atenção Primária à Saúde (APS). Este estudo abrange a avaliação de múltiplos indicadores de qualidade, recomendados por Grupo Técnico Consultivo de Especialistas em Vigilância Nutricional (TEAM) da Organização Mundial de Saúde (OMS) e do Fundo das Nações Unidas para Infância (Unicef)^19^. Espera-se com este trabalho orientar os envolvidos em ações de VAN sobre como melhorar a qualidade dos dados antropométricos, visando oferecer maior confiabilidade às métricas para o monitoramento local, estadual e nacional do estado nutricional da população infantil brasileira.

## MÉTODOS

### Desenho de Estudo

Trata-se de um estudo descritivo sobre a avaliação da qualidade dos dados antropométricos de crianças de 0 a 59 meses de idade, atendidas nos serviços de APS do Brasil, no período entre 2008 e 2017. As informações foram obtidas a partir das bases de dados individuais e anonimizados do Sisvan.

Os dados brutos do Sisvan, disponibilizados para uso neste projeto pelo Centro de Integração de Dados e Conhecimentos para Saúde (Cidacs), Fundação Oswaldo Cruz (Fiocruz), foram utilizados em conformidade com os protocolos institucionais de segurança e privacidade de dados e conforme estabelece a Resolução 466/2012 da Comissão Nacional de Ética em Pesquisa do Conselho Nacional de Saúde. O projeto foi submetido e aprovado pelo comitê de ética do Instituto de Saúde Coletiva da Universidade Federal da Bahia (CAAE: 41695415.0.0000.5030).

### Fonte de Dados

As bases de dados do Sisvan são compostas pelos registros do acompanhamento nutricional e alimentar provenientes dos Sistemas de Informação em Saúde da APS. Em relação ao estado nutricional, as bases de dados do Sisvan incluem dados antropométricos registrados no Sistema de Gestão do Programa Bolsa Família, no e-SUS APS e no Sisvan^20^. Os dados referentes ao acompanhamento do estado nutricional dos beneficiários do Programa Bolsa Família (PBF), que ocorre no mínimo duas vezes por ano, são incorporadas ao Sisvan no final de cada vigência do PBF (primeira vigência de janeiro a junho e segunda vigência de julho a dezembro). Os registros provenientes do e-SUS APS são incorporados gradativamente ao Sistema de Informação em Saúde para a Atenção Básica, respeitando o cronograma de envio de dados pelas equipes de saúde, e posteriormente exportados para o Sisvan após processamento e validação dos dados^20^.

### Indicadores de Qualidade dos Dados Antropométricos

A qualidade dos dados antropométricos foi avaliada por meio de múltiplos indicadores, recomendados pela OMS-Unicef^19^: 1) completude (cobertura da população-alvo, completude da data de nascimento e completude das medidas antropométricas); 2) razão entre sexos; 3) distribuição da idade (histogramas da idade em anos/meses e do mês de nascimento; e índice de dissimilaridade da idade em meses); 4) preferência por dígitos de estatura e peso (histogramas dos dígitos terminais e dos números inteiros; e índice de dissimilaridade dos dígitos terminais); 5) valores de escore-z implausíveis (percentual de escores-z implausíveis); 6) desvio-padrão dos escores-z; e 7) normalidade dos escores-z (histogramas, assimetria e curtose). Para análise dos indicadores foram utilizados os dados de sexo, data de nascimento, idade (meses e anos), as medidas de estatura (cm) e peso (kg), bem como os escores-z dos índices antropométricos comumente utilizados na avaliação do estado nutricional de crianças: estatura/idade (E/I), peso/idade (P/I), peso/estatura (P/E) e índice de massa corporal/idade (IMC/I). Os escores-z foram calculados utilizando a ferramenta “STATA igrowup package” e as curvas de referência de crescimento infantil da OMS^21^. Os indicadores de qualidade estimados encontram-se descritos em detalhes no Quadro 1.

**Quadro 1:** Descrição dos indicadores de qualidade dos dados antropométricos. Crianças 0-59 meses de idade. Sisvan, Brasil, 2008-2017.

Indicador de qualidade	Detalhamento do indicador	Dado utilizado
Completude		
Cobertura da população-alvo, usuária do SUS (%)	Número de crianças com registro de estado nutricional no SISVAN dividido pelo número de crianças menores de cinco anos usuárias do SUS, multiplicado por 100. A população usuária do SUS foi obtida pela subtração da população total (estimada pelo IBGE) pela população coberta por plano privado de saúde (obtida a partir da ANS).	Todas as crianças com registro de estado nutricional no SISVAN. Um único registro por criança a cada ano (última data de acompanhamento) foi considerado para o cálculo da cobertura.
Cobertura da população total, residente (%)	Número de crianças com registro de estado nutricional no SISVAN dividido pelo número total de crianças menores de cinco anos (estimada pelo IBGE), multiplicado por 100.	Um único registro por criança a cada ano (última data de acompanhamento) foi considerado para o cálculo da cobertura.
Completude da data de nascimento (%)	Número de registros com dia, mês e ano de nascimento completos dividido pelo número total de registros de crianças no SISVAN, multiplicado por 100.	Todos os registros de acompanhamento do estado nutricional no SISVAN.
Completude das medidas antropométricas (%)	Número de registros com medidas de peso e estatura preenchidas dividido pelo número total de registros de crianças no SISVAN, multiplicado por 100.	Todos os registros de acompanhamento do estado nutricional no SISVAN.
Razão entre sexos		
Razão entre sexos	Número de meninos dividido pelo número de meninas. A razão esperada é aquela representada pela população nacional.	Todos os registros de acompanhamento do estado nutricional no SISVAN.
Distribuição da idade		
Histogramas da idade (em anos, meses e mês de nascimento)	Histogramas foram utilizados para avaliar o padrão de distribuição da idade em meses, em anos e do mês de nascimento. Uma distribuição aproximadamente uniforme é esperada. A idade da criança foi calculada a partir da data de nascimento e data do acompanhamento do estado nutricional.	Todos os registros com mês e ano de nascimento completos.
Índice de dissimilaridade da idade (em meses)	O índice de Myers “*unblended*” foi analisado, conforme fórmula abaixo, para identificar a porcentagem de registros desviando de uma distribuição uniforme de idade em meses (0-59). $\frac{\sum_{i=1}^{60}\left|X_{is}-X_{ie}\right|}{2}$ Em que, X_is_ é a porcentagem observada e X_ie_ é a porcentagem esperada. O índice varia de 0 a 90, sendo 0 o valor ideal.	Todos os registros com mês e ano de nascimento completos.
Preferência por dígitos de estatura e peso		
Histogramas dos dígitos terminais e dos números inteiros para estatura (cm) e peso (kg)	Histogramas foram utilizados para avaliar o padrão de distribuição dos dígitos terminais e números inteiros de estatura e peso. Uma distribuição aproximadamente uniforme é esperada.	Todos os registros com informação de peso e/ou estatura.
Índice de dissimilaridade dos dígitos terminais para estatura (cm) e peso (kg)	O índice de Myers “unblended” foi analisado, conforme fórmula abaixo, para identificar a porcentagem de registros desviando de uma distribuição uniforme de dígitos terminais de estatura e peso. $\frac{\sum_{i=1}^{10}\left|X_{i5}-X_{ie}\right|}{2}$ Em que, X_is_ é a porcentagem observada e X_is_ é a porcentagem esperada. O índice varia de 0 a 90, sendo 0 o valor ideal.	Todos os registros com informação de peso e/ou estatura.
Valores de escores-z implausíveis		
Escores-z implausíveis para cada índice antropométrico (%)	Número de registros com valores de escore-z implausível dividido pelo número total de registros de crianças no SISVAN, multiplicado por 100. Utilizando o sistema de sinalização do macro “STATA igrowup package” da OMS, valores de escore-z implausíveis foram detectados segundo os critérios de plausibilidade (WHO, 1995): E/I (-6, +6), P/E (-5, +5), P/I (-6, +5) e IMC/I (-5, +5). Percentuais acima de 1% é indicativa de baixa qualidade dos dados.	Todos os registros com informação de data de nascimento, peso e/ou estatura.
Dispersão dos valores de escore-z		
Desvio padrão dos valores de escore-z plausíveis para cada índice antropométrico	Desvio padrão foi calculado mediante a fórmula abaixo. $\sqrt{\frac{\sum_{i=1}^{n}{\left(Y_{i}-\bar{Y}\right)}^{2}}{n-1}}$ Em que, n é o número total de observações, Y_i_ é cada valor na base de dados, e *Y*ˉ é a média das observações.	Todos os registros com valores de escore-z biologicamente plausíveis para o índice antropométrico de interesse.
Normalidade dos valores de escore-z		
Distribuição dos valores de escore-z plausíveis para cada índice antropométrico	Gráficos de densidade de Kernel foram usados para examinar o padrão de distribuição dos valores de escore-z para cada índice.	Todos os registros com valores de escore-z biologicamente plausíveis para o índice antropométrico de interesse.
Assimetria dos valores de escore-z plausíveis para cada índice antropométrico	Assimetria foi calculada pelo coeficiente de Fisher-Pearson: $\frac{\sum_{i=1}^{n}{\left(Y_{i}-\bar{Y}\right)}^{3}/n}{s^{3}}$ Em que, *Y*ˉ = média, s = desvio padrão (calculado com ‘n’ no denominador em vez de n-1) e n= tamanho da amostra. Em geral, aceita-se que um coeficiente <-0,5 ou >0,5 indique assimetria.	Todos os registros com valores de escore-z biologicamente plausíveis para o índice antropométrico de interesse.
Curtose dos valores de escore-z plausíveis para cada índice antropométrico	Curtose foi calculada pelo coeficiente de Fisher-Pearson: $\frac{\sum_{i=1}^{n}{\left(Y_{i}-\bar{Y}\right)}^{4}/n}{s^{4}}$ Em que, *Y*ˉ = média, s = desvio padrão (calculado com ‘n’ no denominador em vez de n-1) e n= tamanho da amostra. Em geral, aceita-se que um coeficiente <2 ou >4 indique curtose.	Todos os registros com valores de escore-z biologicamente plausíveis para o índice antropométrico de interesse.

Sisvan: Sistema de Vigilância Alimentar e Nutricional; SUS: Sistema Único de Saúde; IBGE: Instituto Brasileiro de Geografia e Estatística; ANS: Agência Nacional de Saúde Suplementar; E/I: Estatura/Idade; P/E: Peso/Estatura; P/I: Peso/Idade; IMC/I: Índice de Massa Corporal/Idade.

### Processamento e Análise dos Dados

Os dados foram analisados utilizando o software Stata versão 15.1 (Stata Corporation, College Station, USA). Uma vez que o mesmo registro pode ser digitado nos diferentes sistemas de informação que compõem o Sisvan, os registros duplicados foram identificados e removidos considerando as seguintes variáveis: código de identificação do indivíduo, data de nascimento, data do acompanhamento, peso e estatura. A duplicidade do registro foi aceita quando todos os valores das variáveis consideradas eram iguais. Os indicadores de qualidade foram descritos anualmente e segundo as seguintes variáveis, quando aplicável: sexo, idade, região e unidade federativa.

## RESULTADOS

### Cobertura da População-Alvo

No total, 73.745.023 registros e 29.852.480 crianças menores de 5 anos foram identificados nas bases de dados sobre estado nutricional do Sisvan entre 2008 e 2017, após a exclusão de 27.167.791 registros duplicados. A cobertura da população-alvo usuária do SUS aumentou de 17,7% em 2008 para 45,4% em 2017 (Tabela 1). Em relação a população total (residente), a cobertura aumentou de 14,3% para 34,6% nesse mesmo período. O aumento da cobertura foi observado em todas as regiões, com destaque para o Nordeste, cuja cobertura foi a maior em todos os anos e a única a alcançar mais que 50% da população-alvo. Entre as unidades federativas, as maiores coberturas foram observadas em Minas Gerais (71,6% em 2017), Paraíba (69,7%), Piauí (69,4%), Ceará (58,9%) e Maranhão (58,9%). Em 2017, somente 11 unidades federativas possuíam cobertura maior que 50% (Tabela 2).

**Tabela 1:** Indicadores de qualidade dos dados antropométricos. Crianças 0-59 meses de idade. Sisvan, Brasil, 2008-2017.

Indicadores	2008	2009	2010	2011	2012	2013	2014	2015	2016	2017
Total de registros e crianças acompanhadas										
Total de crianças	2.172.824	2.752.361	3.339.585	3.263.701	3.189.390	4.216.064	4.343.156	4.943.025	5.134.115	5.097.377
Total de registros únicos	3.612.015	5.134.933	5.987.400	6.446.453	6.252.667	7.894.213	7.822.944	9.756.986	10.357.127	10.480.285
Total de registros duplicados	1.969.446	2.908.094	2.950.561	2.888.367	2.595.380	2.287.606	2.197.925	2.988.357	3.172.167	3.209.888
Completude										
Cobertura da população-alvo, usuária SUS (%)	17,7	22,9	28,8	28,6	28,6	38,5	39,9	44,7	45,9	45,4
Cobertura da população total, residente (%)	14,3	18,3	22,5	22,2	21,8	28,9	29,8	33,7	34,9	34,6
Completude da data de nascimento (%)	99,9	99,9	99,9	99,9	99,9	99,9	99,9	100	100	100
Completude das medidas antropométricas (%)	100	99,9	100	100	100	100	100	100	98,8	100
Razão entre sexos										
Razão entre sexos	1	1	1	1	0,8	0,8	0,9	1	1	1
Distribuição da idade										
Índice de dissimilaridade da idade em meses (%)	20,4	14,1	12,6	11,9	11,2	10,3	9,7	4,8	1,5	3,8
Preferência por dígitos de estatura e peso										
Índice de dissimilaridade dos dígitos terminais de estatura (%)	88,8	88	88,2	87,9	87,7	89,7	89,9	89,4	88,3	88,8
Índice de dissimilaridade dos dígitos terminais de peso (%)	50,2	47	47,6	46,1	44,3	45,3	44,2	42,8	40,1	40,4
Valores de escores-z implausíveis										
Escores-z implausíveis de E/I (%)	3	3,4	3,8	3,4	3	3,3	3,5	3	2,9	1,9
Escores-z implausíveis de P/E (%)	2,4	2,4	2,7	2,5	2,5	3,3	3,2	3	2,4	2,2
Escores-z implausíveis de P/I (%)	2,1	1,6	1,9	1,8	1,7	2,5	2,4	2,1	1,2	0,8
Escores-z implausíveis de IMC/I (%)	3,9	4,1	4,6	4,2	3,9	4,8	4,8	4,2	3,1	2,9
Dispersão dos valores de escore-z										
Desvio-padrão dos escores-z de E/I	1,5	1,6	1,6	1,6	1,6	1,6	1,6	1,6	1,6	1,6
Desvio-padrão dos escores-z de P/E	1,3	1,4	1,5	1,5	1,5	1,5	1,5	1,5	1,5	1,5
Desvio-padrão dos escores-z de P/I	1,2	1,3	1,3	1,3	1,3	1,3	1,3	1,3	1,3	1,3
Desvio-padrão dos escores-z de IMC/I	1,4	1,5	1,5	1,5	1,5	1,5	1,5	1,5	1,5	1,5
Normalidade dos valores de escore-z										
Assimetria dos escores-z de E/I	0,1	0,2	0,2	0,2	0,1	0,2	0,2	0,1	0,1	0,1
Assimetria dos escores-z de P/E	0	-0,1	-0,1	-0,1	-0,1	-0,1	-0,1	-0,1	0	0
Assimetria dos escores-z de P/I	0	0,1	0,1	0,1	0,1	0,1	0,1	0,1	0,1	0,1
Assimetria dos escores-z de IMC/I	-0,1	-0,1	-0,1	-0,1	-0,1	-0,1	-0,1	-0,1	-0,1	-0,1
Curtose dos escores-z de E/I	4,7	4,5	4,5	4,5	4,5	4,5	4,5	4,4	4,5	4,5
Curtose dos escores-z de P/E	3,9	3,8	3,8	3,8	3,8	3,7	3,7	3,7	3,7	3,7
Curtose dos escores-z de P/I	4,3	4,1	4,1	4,1	4,1	4,1	4,1	4,1	4,1	4,1
Curtose dos escores-z de IMC/I	3,9	3,8	3,7	3,7	3,7	3,7	3,7	3,7	3,7	3,7

Sisvan: Sistema de Vigilância Alimentar e Nutricional; E/I: Estatura/Idade; P/E: Peso/Estatura; P/I: Peso/Idade; IMC/I: Índice de Massa Corporal/Idade.

**Tabela 2:** Coberturas da população-alvo e da população total segundo região e unidade federativa. Crianças 0-59 meses de idade. Sisvan, Brasil, 2008-2017.

Regiões e Estados	População-alvo (usuária SUS)		População total (residente)	
	
	2008	2017	2008	2017
Norte	15,5	47,8	14,3	43,2
Acre	11,7	49,9	11,3	47,9
Amapá	8,4	31	7,7	28,5
Amazonas	15,7	51,1	14	45,5
Pará	15,3	50	14,1	45
Rondônia	15	29,7	14,1	26,9
Roraima	16,6	35,2	15,8	33,5
Tocantins	22,5	58,8	21,5	54,3
Nordeste	23,2	56,7	21,3	49,1
Alagoas	23,6	56,9	21,8	49,4
Bahia	23,2	54,7	21,4	48,6
Ceará	27,9	58,9	25,1	48,3
Maranhão	21,8	58,9	21,1	54,7
Paraíba	32,9	69,7	30,4	60,9
Pernambuco	16,5	49,4	14,5	41,5
Piauí	24,8	69,4	23,2	59,8
Rio Grande do Norte	23,5	47,6	20,6	39,3
Sergipe	17,4	55,3	15,7	46,3
Centro-Oeste	10,5	30,7	9,2	24,2
Distrito Federal	0,9	13,3	0,7	9,7
Goiás	12,6	27,9	11,3	22,3
Mato Grosso	11	38,6	10	31,1
Mato Grosso do Sul	14,1	41,8	12,3	33
Sudeste	14,2	38,5	9,5	25,1
Espírito Santo	24,1	31,5	18,7	23,4
Minas Gerais	19,5	71,6	15,5	51,7
Rio de Janeiro	9,7	26,8	6,6	17,8
São Paulo	11,6	27,1	7	16,5
Sul	17,8	42,3	14,2	31,4
Paraná	19,9	48	15,9	34,6
Rio Grande do Sul	16,3	35,6	13,1	26,7
Santa Catarina	16,4	43,3	13,1	33,2
Brasil	17,7	45,4	14,3	34,6

Sisvan: Sistema de Vigilância Alimentar e Nutricional. SUS: Sistema Único de Saúde.

### Completude da Data de Nascimento e de Medidas Antropométricas

O percentual de registros com data de nascimento completa no Sisvan foi alta para todo o período estudado, variando de 99,9% em 2008 a 100% em 2017 (Tabela 1). O percentual de registros com as medidas de peso e altura preenchidas também foi alto, apresentando 100% para quase todos os anos (Tabela 1).

### Razão entre Sexos

A razão entre sexos variou entre 0,8 e 1 ao longo dos anos. A variabilidade nesse indicador foi identificada entre 2012 e 2014, quando as razões mostravam maior número de meninas em relação a meninos (Tabela 1).

### Distribuição da Idade

Os histogramas da idade em anos completos revelam um padrão de maiores percentuais de registros entre as idades de 2 a 4 anos até meados de 2015, quando a distribuição da idade se tornou mais uniforme (Figura 1a). Esse padrão foi observado principalmente nas regiões Norte e Nordeste. Os meses de nascimento apresentaram distribuição aproximadamente uniforme, independente do ano, sexo e região (Figura 1b). Segundo o índice de dissimilaridade, o percentual de registros que precisariam ser redistribuídos para obter uma distribuição uniforme da idade em meses reduziu de 20,4% em 2008 para 3,8% em 2017 (Tabela 1).

**Figura 1:** Distribuição da idade em anos (a) e do mês de nascimento (b) na população total (1) e segundo sexo (2) e região (3). Crianças 0-59 meses de idade. Sisvan, Brasil, 2008-2017.

### Preferência por Dígitos para Estatura e Peso

Os histogramas da Figura 2a mostram preferência de quase 100% pelo dígito terminal zero para estatura e uma preferência dos dígitos terminais zero e cinco para peso. Quase 90% dos registros de estatura (maior número possível) precisariam ser redistribuídos para obter uma distribuição uniforme dos dígitos terminais em todos os anos; enquanto para os dígitos terminais de peso, o percentual de registros que precisariam ser redistribuídos reduziu de 50,2% em 2008 para 40,4% em 2017 (Tabela 1). A distribuição dos números inteiros apresentou vários picos perceptíveis para medida de estatura (por exemplo 100 cm) (Figura 2a), enquanto a distribuição dos números inteiros para peso foi muito adequada, não apresentando qualquer pico (Figura 2b).

**Figura 2:** Preferência por dígitos terminais (a) e por números inteiros* (b) de estatura em cm (1) e peso em kg (2). Crianças 0-59 meses de idade. Sisvan, Brasil, 2008-2017.

* Os números inteiros representados no eixo x (horizontal) dos gráficos incluem 40-140 cm para estatura (b.1) e ≤ 30 kg para peso (b.2).

### Valores de Escore-z Implausíveis

Os percentuais de escores-z implausíveis, segundo os pontos de corte da OMS, variaram até meados de 2014, quando uma redução foi observada nos anos seguintes para todos os índices antropométricos (Tabela 1): E/I (3,5% em 2014 *vs*. 1,9% em 2017), P/E (3,2% *vs*. 2,2%), P/I (2,4% *vs*. 0,8%) e IMC/I (4,8% *vs*. 2,9%). Os maiores percentuais de implausibilidade foram identificados entre os registros de crianças menores de 2 anos e das regiões Norte, Nordeste e Centro-Oeste (Figura 3).

**Figura 3:** Percentual de escores-z implausíveis para estatura/idade (a), peso/estatura (b), peso/idade (c) e IMC/idade (d) segundo sexo (1), idade em anos completos (2) e região de residência (3). Crianças 0-59 meses de idade. Sisvan, Brasil, 2008-2017.

### Dispersão dos Valores de Escore-z

Valores de desvio-padrão superiores a 1 para as medidas plausíveis de escore-z foram encontrados para todos os índices antropométricos em todo período (Tabela 1). Os menores valores de desvio-padrão foram observados em 2008. Nota-se uma estabilidade na dispersão dos escores-z para todos os índices após 2008: E/I (1,6), P/E (1,5), P/I (1,3) e IMC/I (1,5). Em geral, houve maior dispersão dos valores de escores-z entre as crianças com 2 anos de idade ou menos e residentes das regiões Nordeste, Norte e Centro-Oeste (Figura 4).

**Figura 4:** Desvio-padrão dos escores-z de estatura/idade (a), peso/estatura (b), peso/idade (c) e IMC/idade (d) segundo sexo (1), idade em anos completos (2) e região de residência (3). Crianças 0-59 meses de idade. Sisvan, Brasil, 2008-2017.

### Normalidade dos Valores de Escore-z

As curvas de distribuição dos escores-z em 2008 e 2017 apresentaram achatamento e desvio à esquerda para E/I, enquanto achatamento mais modesto e desvios à direita foram observados nas distribuições dos escores-z de P/E, P/I e IMC/I, comparando ao padrão de distribuição normal das curvas de crescimento infantil da OMS (Figura 5). Segundo os valores do coeficiente de Fisher-Pearson, a distribuição dos escores-z foi simétrica para todos os índices, enquanto que as distribuições dos escores-z de E/I e P/I apresentaram curtose do tipo platicúrtica em todos os anos, explicado pelos coeficientes acima de 4 e achatamento da curva (Tabela 1).

**Figura 5:** Gráficos de densidade de Kernel dos escores-z de estatura/idade (a), peso/estatura (b), peso/idade (c) e IMC/idade (d) em 2008 (1) e 2017 (2). Crianças 0-59 meses de idade. Sisvan, Brasil, 2008-2017.

## DISCUSSÃO

Este estudo examina a qualidade dos dados antropométricos de crianças atendidas na APS entre 2008 e 2017. Trata-se do primeiro estudo a avaliar a qualidade dos dados individuais do Sisvan no Brasil, abrangendo múltiplos indicadores e diferentes dimensões. De forma geral, os resultados mostram que a qualidade dos dados antropométricos coletados e registrados nos sistemas de informação da APS tem melhorado substancialmente ao longo dos anos. Esses achados representam um marco para consolidação do Sisvan, permitindo melhor clareza e confiabilidade no uso e análise dos dados para identificação dos agravos nutricionais na população e tomada de decisões no âmbito das políticas de alimentação e nutrição.

A completude é uma das dimensões da qualidade de dados, que está diretamente relacionada a vieses de seleção e consequentemente à representatividade dos resultados. Observou-se em todo período alta completude da data de nascimento e das medidas antropométricas, que são informações obrigatórias para realização do registro. Os resultados também mostram uma crescente expansão das coberturas do Sisvan ao longo dos anos, sobretudo entre a população usuária do SUS. Essa expansão pode ser atribuída a importantes avanços: a articulação bem-sucedida das ações de VAN com outras políticas de educação e assistência social (por exemplo, o Programa Saúde na Escola e o PBF^7^); a ampliação da cobertura de agentes comunitários de saúde e equipes de saúde da família^11^; a ampliação e qualificação da APS, por meio dos Núcleos de Apoio a Saúde da Família e do Programa de Melhoria do Acesso e da Qualidade da Atenção Básica (PMAQ-AB)^7^; e os investimento nas ações de VAN, por meio do Financiamento das Ações de Alimentação e Nutrição (FAN) e do apoio financeiro destinado aos municípios para aquisição de equipamentos antropométricos adequados na atenção primária^22^.

Apesar do nítido avanço ao longo dos anos, a cobertura do Sisvan ainda permanece incipiente para maioria das regiões e unidades federativas do país. Em 2017, somente 11 das 27 unidades federativas possuíam cobertura acima de 50%. Das 11 unidades federativas, nove são da região Nordeste, que concentra o maior número de beneficiários do PBF^23^. Estudos prévios mostram que cerca de 80% dos registros antropométricos incorporados pelo Sisvan, entre 2008 e 2013, eram provenientes das condicionalidades do PBF^11^, que inclui o monitoramento nutricional de crianças menores de 7 anos, refletindo em maiores coberturas do Sisvan para a população mais socioeconomicamente vulnerável do país. Vale destacar que, devido à pandemia de covid-19 e seus efeitos sobre os serviços de saúde, uma queda substancial no acompanhamento das condicionalidades do PBF foi detectada em 2020. Das 7,3 milhões de crianças beneficiárias, aproximadamente 2 milhões tiveram registro de acompanhamento, o que corresponde a uma cobertura de somente 25,5%^24^.

Outro aspecto importante relacionado à validade externa e representatividade dos dados é a distribuição da população segundo sexo e idade. A razão entre sexos encontrada no Sisvan foi equilibrada e aproximadamente similar à esperada para população brasileira menor de 5 anos de idade (1,05)^25^. A distribuição da idade em anos e meses completos não apresentou picos substanciais. No entanto, um padrão de maiores percentuais de registros entre as idades de 2 a 4 anos foi observado até meados de 2015, quando a distribuição da idade se tornou mais uniforme. Esse padrão foi observado principalmente nas regiões Norte e Nordeste. Como essas regiões concentram o maior número de registros no Sisvan, sendo eles provenientes do PBF^11^, parte dessa distribuição da idade pode ser explicada pela captação e acompanhamento tardios das crianças pelo programa.

A preferência por dígitos de estatura e peso pode sinalizar desde o arredondamento de medidas até o uso de equipamentos e cuidados inadequados durante a coleta e registro dos dados. Observou-se preferência dos dígitos terminais zero e cinco nas medidas de estatura e peso, indicando erro sistemático pelo arredondamento das medidas. Entre os números inteiros, vários picos perceptíveis para medida de estatura foram observados (por exemplo, 100 cm), revelando possíveis problemas com equipamento ou arredondamento das medidas. Por outro lado, a distribuição dos números inteiros para peso foi muito adequada.

Embora críticos para obtenção de prevalências precisas do estado nutricional, esses resultados são relativamente comuns e esperados, especialmente para as medidas de estatura^8^. Na maioria das réguas antropométricas, as marcas de centímetros são maiores e mais fáceis de ler do que as de milímetros, induzindo as equipes menos diligentes ou menos instruídas a registrar os valores arredondados. Como observado nos dados do Sisvan, o arredondamento das medidas de peso foi menos comum, devido possivelmente ao uso de balanças digitais cujo displays fornecem valores numéricos com decimais de fácil leitura.

Vale ainda ressaltar a adequação das estruturas e equipamentos para coleta desses dados nas unidades básicas de saúde. De acordo com um estudo recente, realizado a partir dos dados da avaliação externa do PMAQ-AB em 2014, somente 35% das unidades básicas de saúde no Brasil possuíam estrutura adequada para desenvolvimento das ações de alimentação e nutrição, incluindo balança para adultos, balança infantil, régua antropométrica, fita métrica e caderneta de saúde da criança^26^.

Os indicadores de implausibilidade, dispersão e normalidade dos escores-z estão normalmente associados a erros de mensuração, data de nascimento imprecisa ou erros no registro dos dados. Apesar da redução de valores implausíveis entre os registros do Sisvan a partir de 2014, os percentuais ainda excediam 1% para todos os índices, sugerindo baixa qualidade dos dados de acordo com o sistema de implausibilidade da OMS^2,19^. Resultado similar foi encontrado para o indicador de dispersão dos escores-z. Embora os valores de desvio-padrão tenham se mantido estáveis ao longo dos anos, notou-se uma grande dispersão dos escores-z para a maioria dos índices. Estudos prévios relatam ampla variação no desvio-padrão de escores-z antropométricos em crianças menores de 5 anos em pesquisas de demografia e saúde em vários países de baixa e média renda^10,18^.

Padrões consistentes de maiores percentuais de implausibilidade e dispersão dos escores-z foram observados entre os índices antropométricos incluindo a medida de estatura e entre os registros de crianças menores de 2 anos de idade e residentes das regiões Norte, Nordeste e Centro-Oeste. Esses resultados apontam para erros e limitações bem conhecidos acerca da coleta e registro de medidas antropométricas. Geralmente, espera-se que o desvio-padrão dos escores-z de E/I e IMC/I seja maior que o dos demais índices, devido a maior dificuldade e chance de erros na coleta das medidas de estatura e idade.

Esse padrão é especialmente esperado no grupo de crianças menores de 2 anos, cujo comprimento/estatura é medido com a criança deitada e a precisão da idade em meses é mais crítica devido à velocidade de crescimento mais acelerada nessa faixa etária^(19,27)^. Além disso, vale destacar que as regiões onde o desvio-padrão e percentual de valores implausíveis foram maiores são as mais vulneráveis do ponto de vista de estrutura adequada para vigilância nutricional nas unidades básicas de saúde, segundo estudo com dados do PMAQ-AB^26^.

Diferentes parâmetros e medidas de normalidade foram utilizados para avaliar a distribuição dos escores-z de cada índice antropométrico. A partir dos gráficos de densidade de Kernel, observamos desvios da distribuição à esquerda para E/I e à direita para P/E, P/I e IMC/I, quando comparado ao padrão de distribuição normal das curvas de crescimento da OMS. Embora as distribuições dos escores-z tenham se mostrado simétricas para os quatro índices, distribuições cúrticas foram identificadas para E/I e P/I (coeficiente Fisher-Pearson > 4).

Apesar desses achados, ainda não existem evidências consistentes para sugerir que a dispersão e o desvio de uma distribuição gaussiana são devidos apenas à qualidade dos dados. A população de referência da OMS, usada para derivar os escores-z, foi restrita a uma população saudável vivendo em condições ambientais favoráveis ao crescimento saudável^28^. Assim, é possível que distribuições incomuns ocorram em populações mais heterogêneas, como em países com grandes desigualdades sociais. A população do Sisvan representa os usuários da APS no Brasil, composta em sua maioria por beneficiários do PBF; ou seja, uma população socioeconomicamente mais vulnerável. As distribuições encontradas em nosso estudo são coerentes com as estimativas de má nutrição dessa população, que revelam persistente prevalência de baixa estatura e crescente carga de sobrepeso e obesidade infantil^29,30^.

Este estudo apresenta algumas limitações. A interpretação de certos indicadores isoladamente pode não ser suficiente para obter conclusões sobre a qualidade dos dados, especialmente para indicadores que levam em consideração a dispersão e distribuição dos escores-z. Mais pesquisas são necessárias para quantificar em termos definitivos quanto da distribuição dos escores-z é atribuído à heterogeneidade da população e a erros de medição. Na ausência de pontos de corte ou abordagens mais apropriadas que levem em conta essas limitações, uma avaliação conjunta dos indicadores de qualidade é recomendada^19^.

Com base nos resultados deste estudo, destaca-se a importância de ações para melhoria dos pontos críticos identificados na qualidade dos dados antropométricos do Sisvan: 1) manutenção e expansão de políticas intersetoriais e programas de saúde que promovam ações de VAN, como ocorrem no PBF, Programa Saúde na Escola e Crescer Saudável; 2) desenvolvimento de ações de qualificação e educação permanente (preferencialmente atentas e flexíveis às diferentes realidades locais); 3) manutenção e ampliação do apoio financeiro aos municípios para estruturação da VAN na APS, por meio da aquisição e calibração periódica de equipamentos antropométricos; 4) informatização nos serviços da APS, permitindo que profissionais nas unidades básicas de saúde prontamente registrem os dados dos atendimentos, incluindo os dados de peso e estatura, no prontuário eletrônico do paciente no e-SUS APS; e 5) implantação e implementação de rotina para verificação e produção contínua de relatórios sobre a qualidade dos dados do Sisvan.

## CONCLUSÕES

Em geral, nossos resultados sugerem que a qualidade dos dados antropométricos do Sisvan tem substancialmente melhorado ao longo dos anos. Entretanto, alguns indicadores ainda requerem atenção. A cobertura da população-alvo permanece incipiente para um sistema de vigilância cujo objetivo é o monitoramento universal do público usuário da atenção primária do SUS. A precisão e a qualidade das medidas antropométricas, sobretudo para estatura, foram inferiores nos registros de crianças menores de 2 anos e residentes das regiões Norte, Nordeste e Centro-Oeste. Esses grupos representam a parcela da população infantil mais vulnerável a agravos nutricionais, necessitando de estimativas precisas que possam subsidiar o monitoramento do perfil nutricional da população e a elaboração de políticas públicas.
